# Switching TNF antagonists in patients with chronic arthritis: an observational study of 488 patients over a four-year period

**DOI:** 10.1186/ar1881

**Published:** 2006-01-06

**Authors:** Juan J Gomez-Reino, Loreto Carmona

**Affiliations:** 1 Rheumatology Service and Department of Medicine, Hospital Clinico Universitario, Medical School, Universidad de Santiago de Compostela, Spain; 2 Unidad de Investigacion, Sociedad Española de Reumatologia, Madrid, Spain; 3 A list of participating investigators and centers appears in Acknowledgements

## Abstract

The objective of this work is to analyze the survival of infliximab, etanercept and adalimumab in patients who have switched among tumor necrosis factor (TNF) antagonists for the treatment of chronic arthritis. BIOBADASER is a national registry of patients with different forms of chronic arthritis who are treated with biologics. Using this registry, we have analyzed patient switching of TNF antagonists. The cumulative discontinuation rate was calculated using the actuarial method. The log-rank test was used to compare survival curves, and Cox regression models were used to assess independent factors associated with discontinuing medication. Between February 2000 and September 2004, 4,706 patients were registered in BIOBADASER, of whom 68% had rheumatoid arthritis, 11% ankylosing spondylitis, 10% psoriatic arthritis, and 11% other forms of chronic arthritis. One- and two-year drug survival rates of the TNF antagonist were 0.83 and 0.75, respectively. There were 488 patients treated with more than one TNF antagonist. In this situation, survival of the second TNF antagonist decreased to 0.68 and 0.60 at 1 and 2 years, respectively. Survival was better in patients replacing the first TNF antagonist because of adverse events (hazard ratio (HR) for discontinuation 0.55 (95% confidence interval (CI), 0.34–0.84)), and worse in patients older than 60 years (HR 1.10 (95% CI 0.97–2.49)) or who were treated with infliximab (HR 3.22 (95% CI 2.13–4.87)). In summary, in patients who require continuous therapy and have failed to respond to a TNF antagonist, replacement with a different TNF antagonist may be of use under certain situations. This issue will deserve continuous reassessment with the arrival of new medications.

## Introduction

When initiated early in rheumatoid arthritis (RA), significant control of joint inflammation and damage and improvement in physical function are obtained with disease modifying antirheumatic drugs (DMARDs), alone or in combination with tumor necrosis factor (TNF) antagonists [1]. Three TNF antagonists, infliximab, etanercept, and adalimumab, have demonstrated efficacy in RA [2-4] and are commercially available. The World Health Organization Collaborating Center consensus proposed that RA patients with active disease who have failed to respond to an adequate course of DMARDs are eligible for anti-cytokine therapy [5]. Other guidelines recommend a similar indication for these agents. In other forms of chronic arthritis, TNF antagonists are also recommended for patients whose disease does not respond to non-steroidal anti-inflammatory drugs or DMARDs [6-9].

In RA, evidence based on clinical trials suggests that these three drugs are equally effective, though they have distinct structural, pharmacokinetic, and pharmacological properties [10], and differences in their modes of action [11]. Comparable effectiveness has also been found in clinical settings [12]. Nevertheless, a proportion of patients do not benefit from treatment with a certain TNF antagonist, and thus the use of a second antagonist when the first has failed is advocated based on a few clinical reports of small numbers of patients [13-16]. For the other forms of chronic arthritis, this information is still lacking; whether a second TNF antagonist would be effective is a relevant clinical question.

In February 2000, the Spanish Society of Rheumatology (SER) launched a registry (Base de Datos de Productos Biológicos de la Sociedad Española de Reumatología (BIOBADASER)) for patients with rheumatic conditions treated with biologics, including TNF antagonists. Over the last four and half years, 4,706 patients from 95 hospitals have been included in this registry and have been actively followed. Although the emphasis of the registry is drug safety, information on discontinuation of TNF antagonists for any cause is gathered as well. In the present study, we analyze the drug survival rates of TNF antagonists, as a surrogate for their effectiveness, in 488 patients with rheumatic diseases who had switched from one TNF antagonist to another.

## Materials and methods

BIOBADASER methodology has been described previously [17] and is detailed the BIOBADASER website [18]. Briefly, BIOBADASER is a registry established in February 2000 for the active long-term follow-up and assessment of the safety of biological response modifiers in rheumatic patients. The registry, which is supported by the SER and funded, in part, by the Spanish Agency for Medicines and Medical Devices, notes relevant adverse events occurring during and after treatment. Patients registered in BIOBADASER are those with rheumatic diseases being treated with any of the approved biological response modifiers in the participating centers; participation is voluntary. Infliximab was made available for clinical use in August 1999, etanercept in April 2003 and adalimumab in September 2003 (some patients actually started on adalimumab before general availability, as part of a clinical study, and their data were entered in BIOBADASER once the study ended as all relevant variables had been collected properly). SER guidelines do not propose molecule-specific criteria for prescribing any of the TNF inhibitors.

Data collected systematically include gender, date of birth, diagnosis, date of diagnosis, treatment type, and dates of commencement and of discontinuation. Should a patient discontinue the treatment, the main reason for stopping is also recorded (inefficacy, adverse event, or other causes). When a patient has a relevant adverse event, additional data are registered, including the date of occurrence, type and classification of event, outcome, concomitant treatment, and co-morbidity. The quality of our database is assured by a clear definition of its aim, an optimized number of variables, and an easy method of data collection that allows consistency checks. Incompleteness and agreement of data with patient charts are assessed in site by annual audits of 10% of all patients registered. All errors are corrected accordingly following these audits, yielding an expected underreporting of 11% of actual discontinuations or adverse events. Data from centers in which there is proved incompleteness of data are censored at the time of the last reliable information. The registry was approved by the Spanish Medicines Agency (Ministerio de Sanidad y Consumo), and the information regarding patients was gathered in the registry according to the present official regulations on data protection. The number of patients receiving biologics for rheumatic diseases registered in BIOBADASER represents around 60% of the total population treated in Spain.

### Statistical methods

The cumulative rate of discontinuation was calculated using the actuarial method with multiple-failure per patient. Survival curves in figures are presented with patients starting each time interval, but not with failures, data which are included in the survival estimates for simplicity. The time of starting the TNF antagonist was time 0. The log-rank test was used to compare survival curves, and Cox regression models were used to assess the difference between groups and to measure association with risk factors for discontinuation. The variables included in the Cox models for discontinuation of first treatment were sex, age group, TNF-antagonist, year of first treatment with a TNF-antagonist, and diagnosis (RA, ankylosing spondylitis, psoriatic arthritis, juvenile idiopathic arthritis, or others). The variables included in the Cox models for discontinuation of the second treatment were the same plus the main reason of discontinuation of the first treatment. The bivariate models included primarily only the dependent variable and one independent variable at a time. Subsequently, all variables reaching a *p *value < 0.10 were included in multivariate models to assess independent associations and better ascertain interactions between variables. Additionally, we wanted to identify the characteristics of patients with longer survival of the second course of treatment (more than one year). For this analysis we used logistic regression, bivariate and multivariate analyses, in which the dependent variable was long duration of the second treatment and the explanatory variables those listed above.

## Results

A total of 3,130 women and 1,576 men using TNF antagonists with an overall age of 50 ± 15 years (mean ± standard deviation) were registered in BIOBADASER from the starting date in February 2000 until September 2004. Diagnoses were 68% RA, 11% ankylosing spondylitis, 10% psoriatic arthritis, and the remaining 11% a variety of other chronic inflammatory rheumatic conditions. There was a total of 5,263 treatments with TNF antagonists, and 1,221 discontinuations. The reasons for discontinuation were adverse events in 562 (46%), inefficacy in 465 (38%), patients' decision in 86 (7%), physicians' decision in 38 (3%), improvement in 14 (1%), pregnancy in 10 (1%), poor vein access in 8 (1%), and renal failure secondary to previously known amyloidosis in 3 (0.5%). Fifteen patients were lost to follow up. A total of 488 patients were treated with more than one TNF antagonist (441 patients with two, and 47 with at least three), of whom 385 had RA. The total exposure rate was 9,269 patient years: 7,109 patient years for infliximab, 1,863 patient years for etanercept, and 295 patient years for adalimumab.

**Figure 1:**
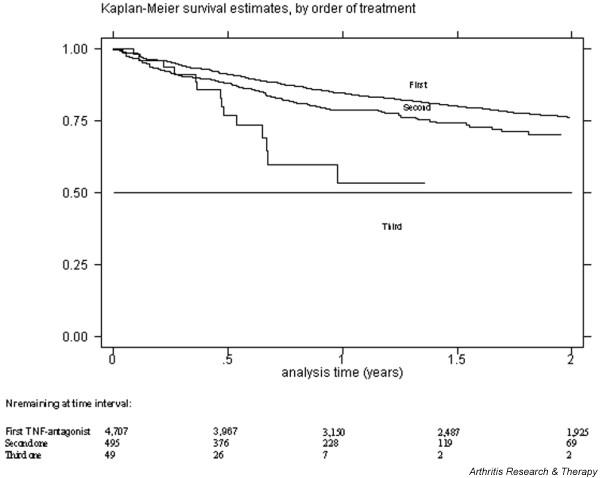
Survival curve of tumor necrosis factor (TNF) antagonists in BIOBADASER during the first two years of use, ranked by order of treatment. The numbers under the curves represent the patients known to be still on treatment by the end of the interval. Differences in the number between intervals do not mean that patients failed (stopped TNF antagonist). They actually represent the patients known to be still on treatment as of the time marked.

Drug survival at first year of treatment depended not only on whether it was the first or successive treatment (Figure 1), but also on the agent used. In this sense, survival of the first TNF antagonist after one year of treatment was slightly lower, but statistically significant, for infliximab (0.81 (range 0.79 to 0.82)) compared to etanercept (0.88 (0.85 to 0.91)) and adalimumab (0.87 (0.82 to 0.91)). The drug survival is consistently lower when the drugs are used as a second treatment, and statistically lower for infliximab (0.34 (0.19 to 0.51)) than for etanercept (0.76 (0.68 to 0.81)) or adalimumab (0.67 (0.42 to 0.83)). However, infliximab treatments were first started 40 and 50 months before any etanercept and adalimumab treatments, respectively. A similar trend was seen for the third TNF antagonist used, but the numbers were too small for a meaningful analysis.

**Figure 2:**
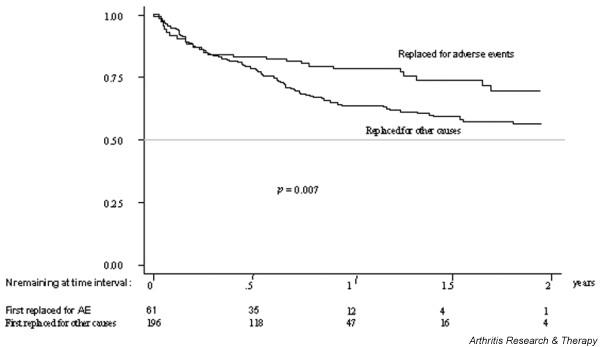
Survival of the second tumor necrosis factor (TNF) antagonist depending on the reason of replacement of first treatment. The numbers under the curves represent the patients known to be still on treatment by the end of the interval. Differences in the number between intervals do not mean that patients failed (stopped TNF antagonist). They actually represent the patients known to be still on treatment as of the time marked. AE, adverse effect.

Reasons for discontinuation of the three TNF antagonists were similar (Table 1); adverse events were the most frequent reason (48%) for discontinuing the agent used in the first place. The kappa value for concordance between the reasons for discontinuation of the first versus the second treatment was 0.29. Survival of the second TNF antagonist was better (*p *= 0.007) if the first one was replaced because of an adverse event (Figure 2).

### Replacement of infliximab by etanercept

There were 356 patients switched from infliximab to etanercept. The first year survival of etanercept was 0.78 (95% confidence interval (CI): 0.71–0.83). The median treatment duration to discontinuation of infliximab was 0.56 (P_25–75_: 0.46–0.68) years, and of etanercept 0.24 (P_25–75_: 0.16–0.34) years (*p *< 0.001). There were no statistical differences in the reasons for discontinuing infliximab or etanercept; these were inefficacy in 57% of the cases and adverse events in 42%.

### Replacement of etanercept by infliximab

Fifty-two patients who failed to respond to etanercept as the first TNF antagonist received infliximab. First year survival of infliximab was 0.28 (95% CI: 0.15–0.42). The median treatment duration to discontinuation of etanercept was 0.51 (95% CI: 0.19–0.84) years, and of infliximab 0.51 (95% CI: 0.19–0.66) years. There were differences, however, in the reasons for discontinuing etanercept and infliximab. Etanercept was discontinued in 82% of the cases because of inefficacy, and infliximab was discontinued due to adverse events in 54% of cases (χ^2 ^*p *= 0.015).

### Replacement of infliximab by adalimumab

Thirty-three patients switched from infliximab to adalimumab. First-year survival of adalimumab was 0.69 (95% CI: 0.43–0.85). The median treatment duration to discontinuation of infliximab was 0.73 (95% CI: 0.04–0.95) years, and this duration was 0.18 (95% CI: 0.12) years for adalimumab. The reasons for discontinuation were similar for the two agents.

### Replacement of etanercept by adalimumab

Only 14 patients switched from etanercept to adalimumab, of whom two had discontinued as of analysis day. First year survival of adalimumab was 0.75 (95% CI: 0.31–0.93). The median treatment duration to discontinuation of etanercept was 0.55 (95% CI: 0.23–1.29) years, and 0.06 (no 95% CI available) years for adalimumab (*p *= 0.003). Reasons for discontinuation were similar for etanercept and adalimumab.

### Risk factors for discontinuation and factors associated with a prolonged second course of treatment

There are no clear differences in the survival of the second TNF antagonist among patients with different diagnosis, although there seems to be a trend toward worse survival in the second trial of a TNF antagonist in RA, and even more in juvenile idiopathic arthritis (Table 2).

There are also no differences in survival at one year depending on the year of start of the first TNF antagonist: 84% in year 2000, 82% in year 2001, 81% in year 2002, and 83% in year 2003. According to the results of the bivariate Cox regression models, the factors with the strongest association with discontinuation of the first treatment were therapy with infliximab (hazard ratio (HR) 1.50 (95% CI: 1.27–1.77)) and a diagnosis of RA (HR 1.36 (95% CI: 1.18–1.56)). We did not find any interaction between diagnosis and individual TNF antagonist. Being older than 60 (HR 1.21 (95% CI: 1.07–1.38)) and female (HR 1.25 (95% CI: 1.10–1.43)) were not independently associated with discontinuation, as demonstrated by the introduction of the diagnosis of RA in the models. Infliximab has the strongest association with discontinuation of the second treatment (HR 3.83 (95% CI: 2.58–5.68)). Having suspended the first treatment as a consequence of adverse events reduced the probability of discontinuation of the second treatment by half (HR 0.54 (95% CI: 0.34–0.84)).

## Discussion

In this study, we analyzed drug survival in patients switching TNF antagonists for chronic arthritis. Overall, our results show that the probability of retaining a second TNF antagonist is lower than that of retaining the first one. Of further interest, probability of survival was influenced by diagnosis, reason for drug replacement, and perhaps the molecule used.

Measuring the effectiveness of drugs through observational databases has some limitations, such as assignment of treatment, patient selection bias, and the absence of a washout period [19]. Nonetheless, drug survival can be taken as a sensible indicator of its effectiveness in the clinical setting, and community-based studies that analyze continuation of treatment with different DMARDs are common in rheumatology [20-25]. Furthermore, withdrawal rates of DMARDs in observational studies are similar to those reported in clinical trials [25]. This type of analysis may also demonstrate the effectiveness of new therapies [26].

In a study of RA conducted in seven Swedish clinical centers, discontinuation rates at 24 months of infliximab and etanercept were 25% and 21%, respectively, in agreement with our results [12]. This observation is in contrast with the 0% discontinuation rate reported after 15 months of treatment with infliximab and etanercept in a university clinic in the USA [27]. Parameters other than efficacy and safety, such as co-morbidity, co-medications [28], costs, availability of other therapies, patients' and physicians' expectations, and adherence to treatment [29], are at play. Adherence is also important in this type of analysis. It is a reflection, among other elements, of the patient's compliance [26], pertinent in the case of molecules with different modes of administration, and of variable costs in the diverse health systems. Whether all these factors explain the dissimilarity in the drug survival of TNF antagonists needs further elucidation. Of note, TNF antagonists have similar survival in the different forms of chronic arthritis.

In a previous study, improvement was reported in 8 of 14 RA patients who switched from infliximab to etanercept or from etanercept to infliximab (6 patients) because of adverse events or lack of efficacy [13]. In another study, improvement was observed in 20 patients replacing etanercept with infliximab [14]. The efficacy and safety of four infusions of infliximab in patients failing to respond to etanercept have been described as well [15]. Finally, improvement in inflammation parameters was seen in 12 of 14 patients switching from infliximab to etanercept in another recent study [16]. Information regarding switching to or from adalimumab is not available yet. In the present study, efficacy based on evaluation of clinical parameters was not investigated. Instead, effectiveness was assessed as the probability of drug survival in a large number of patients. Our results indicate that switching TNF antagonists may be effective in a selected group of patients.

Older age emerged as a predictor of shortened drug survival. This is not surprising in light of older patients' recognized risk of suffering medication-related problems [29].

**Figure 3:**
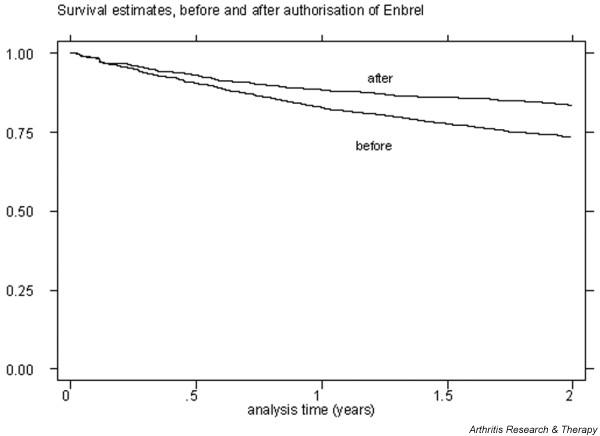
Survival estimates of infliximab before and after the marketing authorization of etanercept (*p *< 0.001).

Unexplained is the lower survival of infliximab when compared to other TNF antagonists, especially when used for replacement therapy. Bias towards the use of new drugs in the most severe or non-responder patients [30] distorts assessment of efficacy. This bias disappears as the pool of patients completes the exposure to the new agent [31]. It should be kept in mind that infliximab treatment was started 40 and 50 months before etanercept and adalimumab, respectively. When infliximab was made available, it was first used in the most severe cases, in those patients in whom good drug survival was not very much expected. As other TNF antagonists became available, patients with a less severe disease were offered these treatments, thus improving overall drug survival (Figure 3). In addition, availability of other TNF antagonists may have led to early drug discontinuation and replacement with a novel agent. In all probability, discontinuation rates of the new TNF antagonists in clinical practice will increase with the arrival of other therapeutic agents. Also, a key variable among the members of the TNF antagonist class is the route of administration [32]. Infusion reaction occurs early in the follow-up of patients with infliximab, which cuts the drug survival dramatically, especially if taking into account that, in this case, the initiation and discontinuation date are the same, something very unusual with other preparations. Furthermore, the intravenous route is generally related to more adverse events. Also, patients may have preferences for particular routes, for example, subcutaneous, and so ask the physician for a change, although this was the main reason for discontinuation in only four cases.

In our study, in contrast with others, differences in cost were not a major consideration for using one or another TNF antagonist, because of the free, unrestricted access to the drugs, provided to all patients by the National Health System.

## Conclusion

Our study supports the replacement of TNF antagonists in a select group of patients with chronic arthropathies who require continuous therapy. Considering the arrival of new medications, this issue will deserve reassessment because of greater expectancies of patients and doctors.

## Abbreviations

BIOBADASER = Base de Datos de Productos Biológicos de la Sociedad Española de Reumatología; CI = confidence interval; DMARD = disease modifying antirheumatic drugs; HR = hazard ratio; RA = rheumatoid arthritis; SER = Spanish Society of Rheumatology; TNF = tumor necrosis factor.

## Competing interests

JJGR is on the Advisory Boards of Wyeth and Roche, and has received lecture fees from Abbott, Wyeth, and Shering Plough.

## Authors' contributions

JJGR prepared the manuscript, which was reviewed and modified by LC. LC planned and ran the analyses. Both were the main designers of the study, helped by other members of the BIOBADASER Study group. The BIOBADASER Study group collected and checked the data without perceiving any economic reward.

## Figures and Tables

**Table 1:** Rate of discontinuation of three tumor necrosis factor antagonists; reasons for discontinuation and rank of treatment

TNF antagonist	Reason for discontinuation	Rate per 100 patient years exposed	
		First treatment	Second treatment

Infliximab	Adverse events	6.5	32.7
	Lack of efficacy	4.7	38.5
			
Etanercept	Adverse events	3.8	6.1
	Lack of efficacy	3.6	9.3
			
Adalimumab	Adverse events	7.2	12.5
	Lack of efficacy	3.2	12.5

TNF, tumor necrosis factor.

**Table 2:** One year drug survival of first and second tumor necrosis factor antagonist, by diagnosis

Diagnosis	Rank of treatment	Number starting	Number failed	Survival (95% CI)
Rheumatoid arthritis	First	2,235	518	0.83 (0.82–0.84)
	Second	194	72	0.79 (0.74–0.83)
				
Ankylosing spondylitis	First	300	49	0.89 (0.86–0.92)
	Second	8	1	0.95 (0.72–0.99)
				
Psoriatic arthritis	First	289	55	0.87 (0.83–0.90)
	Second	15	8	0.81 (0.65–0.90)
				
Juvenile idiopathic arthritis	First	126	16	0.90 (0.84–0.94)
	Second	7	7	0.49 (0.22–0.72)

All differences between survival curves in first and second treatment for each diagnosis have a *p *> 0.001.
